# Is there any association between fluoride exposure and thyroid function modulation? A systematic review

**DOI:** 10.1371/journal.pone.0301911

**Published:** 2024-04-09

**Authors:** Maria Karolina Martins Ferreira, Priscila Cunha Nascimento, Leonardo Oliveira Bittencourt, Giza Hellen Nonato Miranda, Nathalia Carolina Fernandes Fagundes, Fatemeh Vida Zahoori, E. Angeles Martinez-Mier, Marília Afonso Rabelo Buzalaf, Rafael Rodrigues Lima

**Affiliations:** 1 Laboratory of Functional and Structural Biology, Institute of Biological Sciences, Federal University of Pará, Belém, Pará, Brazil; 2 School of Health and Life Sciences, Teesside University, Middlesbrough, United Kingdom; 3 Department of Dental Public Health and Dental Informatics, Indiana University School of Dentistry, Indianapolis, Indiana, United States of America; 4 Department of Biological Sciences, Bauru Dental School, University of São Paulo, Bauru, São Paulo, Brazil; Isawiya General Hospital, Governorate of Gurayyat, SAUDI ARABIA

## Abstract

Numerous pre-clinical and observational studies have explored the potential effects of fluoride (F) at varying concentrations on diverse systems and organs. While some have assessed the endocrinological conditions of children and adults, a consensus regarding the interaction between F and the thyroid remains elusive. This systematic review aimed to gather primary evidence on the association between F and changes in the thyroid at optimal and high levels in water supply as stipulated by the World Health Organization. A search strategy, incorporating terms pertinent to the studies, was employed across PubMed, Scopus, Web of Science, Lilacs, and Google Scholar. Following the review of studies, data were extracted and analyzed using the Grading of Recommendations, Assessment, Development, and Evaluations to assess the quality of the evidence. Our results yielded 3,568 studies, of which seven met the inclusion criteria for this review. Five of the seven studies identified an association between high F exposure and thyroid function. In the analysis of methodological quality, every study was found to have major or minor methodological issues and significant risk of bias. The overall confidence in the evidence was deemed low for all outcomes in the seven studies. The evidence compiled in this review suggests a potential association between chronic high levels of F exposure and thyroid damage. Nonetheless, further studies with robust design and high methodological quality are required to provide evidence for policy makers and health care practitioners.

## Introduction

Fluoride (F) is widely recognized for its role in the prevention and management of dental caries. Initially, it was postulated that the effects were mediated through the systemic ingestion of F, leading to its incorporation into the mineral component of the enamel, apatite [1, 2]. However, subsequent evidence has demonstrated that the mechanisms underlying F’s effects are primarily attributable to the low and constant levels present in the oral cavity, which topically protect the enamel by reducing demineralization and promoting remineralization [3]. The World Health Organization (WHO) has issued recommendations for F levels in the water supply, taking into account the balance between adequate caries protection and minimal risk of dental fluorosis [4, 5]. The latest WHO recommendation stipulates that the addition of F to water should result in a final ion concentration ranging between 0.5 mg/L and 1.0 mg/L. However, it is often reported that the concentrations may fluctuate between 0.7 mg/L and 1.2 mg/L, depending on the local mean temperature. Levels exceeding these recommendations have been associated with detrimental effects on quality of life and the onset of chronic metabolic and bone diseases, such as skeletal fluorosis [6, 7].

While there is no consensus on the association between F levels exceeding the WHO’s recommendations and toxicity, some epidemiological studies have suggested that water fluoridation may pose a threat to public health. On the other hand, it is crucial to acknowledge that humans are typically exposed to F through various sources. These include food and beverages, as well as water from regions with naturally high F levels or from industrialized areas with environmental pollution, thereby indicating a co-exposure scenario. In a recent systematic review and meta-analysis conducted by our group, we found no evidence to suggest that exposure at optimal F levels is associated to neurological damage [8]. However, other organs and systems, such as the thyroid, have been linked to deleterious effects [9, 10].

The thyroid gland plays a pivotal role in organ development and communication, thereby maintaining the homeostatic control of the organism [11]. One such critical component is the thyroid stimulating hormone (TSH), produced by the pituitary gland. TSH serves as the primary growth regulator from the end of fetal life through to adulthood [12]. Modulation of TSH levels can trigger several systemic changes in the organism, including hypothyroidism and hyperthyroidism, both of which are closely associated with energy expenditure and body weight [13]. *In vivo* studies have demonstrated that F can inhibit proteinase activity, responsible for cleaving thyroglobulin into thyroxine (T3) and triiodothyronine (T4). Additionally, F has been shown to cause DNA damage and abnormal increases in parathormone levels (PTH) [11]. Observational studies have corroborated these findings, noting that in areas endemic of fluorosis, elevated F levels were associated with increased TSH levels, decreased T4 and T3 levels, and the onset of morphological changes [14, 15].

Indeed, the relationship between thyroid function and F remains unclear. A systematic review that analyzed the potential association between F exposure and hypothyroidism was conducted by Chaitanya, Karunakar [16]. However, the results lack precision due to the methodological design limitations of the selected studies. The review did not assess the risk of bias or the methodological quality of the included studies. Additionally, the aforementioned review did not adhere to the Preferred Reporting Items for Systematic Review and Meta-Analysis (PRISMA) guidelines, nor did it establish a range of F levels based on WHO recommendations. This omission hindered the generalizability of findings and the comparison of studies. For studies involving environmental pollutants, it is crucial to detail the sampling methods and while discussing the results from primary studies, go beyond the description of its outcomes, and discuss the appropriateness of the methods used to achieve them. These points were not addressed in the Chaitanya, Karunakar [16] study, underscoring the need for a new systematic review with a methodological design that meets the rigor of the scientific method. Therefore, the objective of this systematic review was to evaluate the potential effects of F on the thyroid, summarizing the existing clinical evidence in the literature, and considering the WHO guidelines for defining low and high F concentrations.

## Methods

### Protocol and registration

The protocol for this systematic review, registered under the code CRD42020200268, is available in the PROSPERO database (https://www.crd.york.ac.uk/PROSPERO/). The review was conducted in accordance with the PRISMA statement guidelines [17] (**S1 Table**).

### Eligibility criteria

The selection of studies was based on the PECO acronym. Observational studies were conducted on the human thyroid gland (Population–P), which was exposed to either high (> 2 mg/L) F levels (Exposition–E) or low/optimal (< 1 mg/L) F levels (Comparison–C) through drinking water, with the aim of identifying thyroid impairment in humans (Outcome–O). No restrictions were placed on the source of fluoridation, with both natural and added sources considered. This strategy sought to answer the question: Is there an association between exposure to high F levels and thyroid damage? The standard levels of F in drinking water were determined based on the WHO guideline value [6], with 0.5–1 mg/L and 2 mg/L of F classified as optimal and high levels, respectively. Animal and *in vitro* studies, case reports, case series, editorials, expert opinions, and review articles were excluded from this review.

### Search strategy and study selection

Systematic searches were conducted in several electronic databases, including PubMed, Web of Science, Scopus, LILACS, and The Cochrane Library. Google Scholar and OpenGrey were also utilized as sources of grey literature. The search was carried out up until January 2023. MeSH terms, keywords, and free terms were suitably adapted for each database using Boolean operators (OR, AND) to formulate the search strategy (**S2 Table**). There were no restrictions based on the year or language of publication. Alerts were established in each database to capture studies published during the progression of this systematic review’s other steps.

Following the electronic database searches, all records were transferred to a reference management software (EndNote®, version X9, Thomson Reuters). Duplicate references were subsequently eliminated. The selection of eligible studies was conducted in two sequential stages. First, articles were chosen based on the analysis of their titles and abstracts. Second, these articles were selected by conducting full-text analysis. At each stage, two reviewers (PCN and MKMF) evaluated the articles. In instances of disagreement, a third reviewer (RRL) was consulted. After the final selection, the reference lists of the included studies were reviewed to identify any additional potential studies.

### Data extraction

Information regarding the author, year, study design, and sample characteristics (source, sample size, and age), along with the F levels in the drinking water, thyroid function diagnostic methods, and primary outcomes, was extracted from the selected studies. If necessary, the authors of the included articles were reached via email to resolve queries or to request supplementary data.

### Risk of bias

The Risk of Bias (RoB) in Non-randomized Studies of Exposures (ROBINS-E) tool [18] was adopted to assess risk of bias among the included studies. Seven domains were covered including: (1) bias due to confounding; (2) bias in selecting participants in the study; (3) bias in exposure classification; (4) bias due to departures from in- tended exposures; (5) bias due to missing data; (6) bias in outcome measurement; (7) bias in the selection of reported results. Each domain was characterized as low, moderate, serious or critical risk of bias.

### Level of evidence

The “Grading of Recommendations, Assessment, Development, and Evaluation” (GRADE) tool was employed to assess the overall certainty of evidence [19]. This was necessary owing to the narrative evidence profile, given the wide variation in assessment methods, age, and F levels across studies. Each included study was evaluated based on risk of bias, inconsistency, indirectness, and imprecision. However, a quantitative GRADE analysis was not feasible owing to significant heterogeneity among the studies in defining high levels of F.

## Results

### Selection and characteristics of included studies

**Fig 1:**
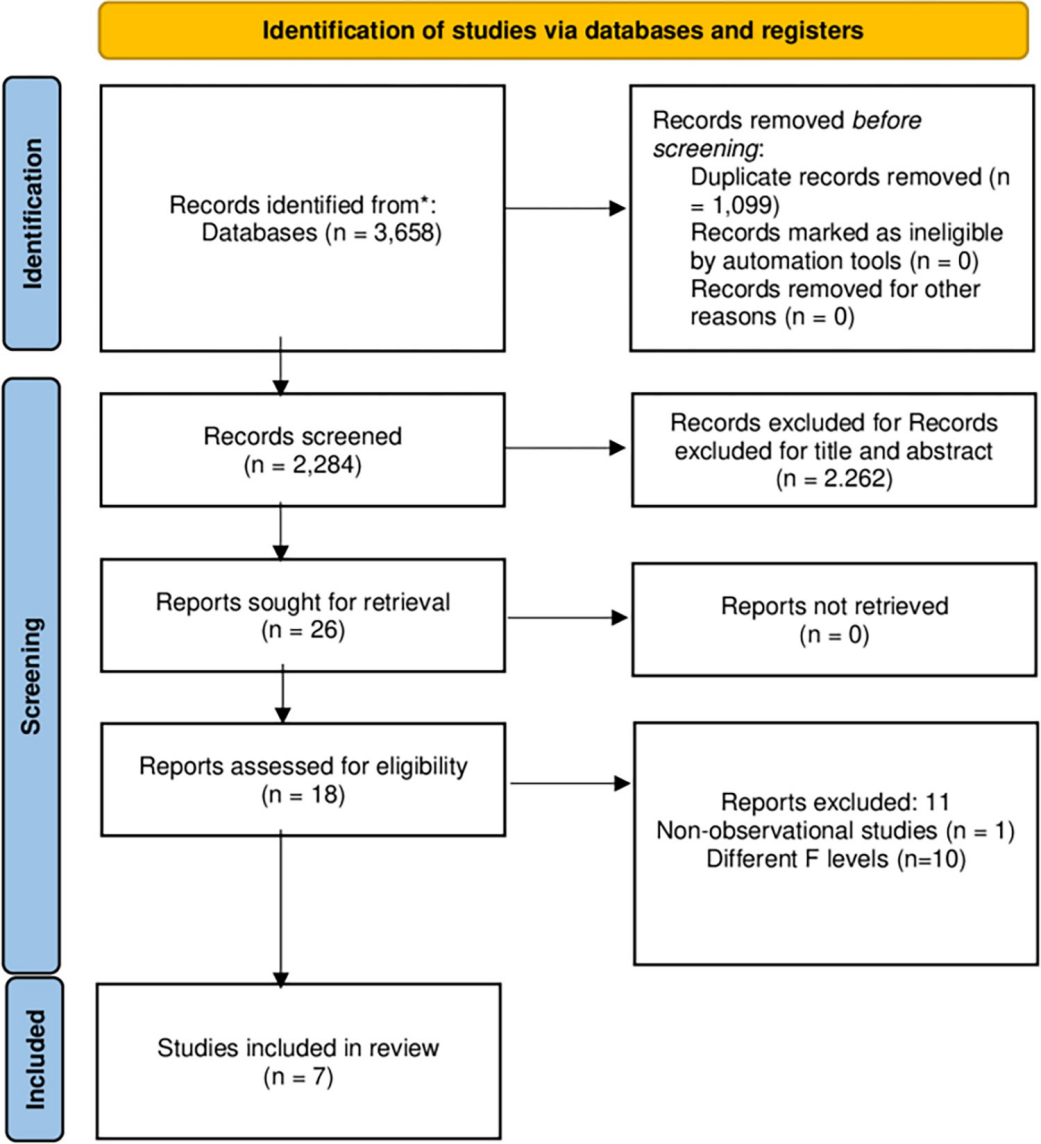
Flow diagram of databases searched according to PRISMA guidelines.

A total of 3,385 articles were identified through database and grey literature searches. After removing duplicates, 1,099 articles were excluded, leaving 2,286 studies. Upon reviewing titles and abstracts, 2,260 studies were further excluded, resulting in 26 studies selected for full-text review. Finally, seven studies were deemed suitable for final consideration and were subjected to qualitative analysis. Fig 1 illustrates the selection process and provides specific reasons for exclusion after full-text review.

### Individual results of the included studies

According to the study design, all the seven selected studies were classified as analytical cross-sectional. The oldest article in this selection was from 1964 [20], followed by [21]. Most of the articles were from Asian countries, such as Pakistan, India, China, and Turkey. Additionally, two articles originated from Europe and North America.

The studies did not categorize participants according to life stages, such as childhood, adolescence, adulthood, middle age, or senior years. Wang, Wang [22], Kutlucan, Koroglu [15], Khandare, Validandi [23], Zulfiqar, Rehman [24] and Zulfiqar, Ajaz [25] evaluated participants ranging in age from 7 to 18 years. Conversely, Leone, Leatherwood [20] focused on adult and older individuals, with ages ranging from 18 to 60 years. Baum, Börner [21], however, did not describe the ages of their participants. Leone, Leatherwood [20], Baum, Börner [21], and Kutlucan, Koroglu [15] reported F levels in drinking water based on previous data from official agencies, rather than conducting their own analyses. The other articles evaluated F levels using an F-specific/ion selective electrode. The mean values for low F levels ranged from 0.09 mg/L to 0.87 mg/L, while the range for higher F concentrations was 2.53 mg/L to 6.23 mg/L. Regarding thyroid function, only Leone, Leatherwood [20] employed the protein bound iodine test on the serum of participants. In contrast, other studies utilized various methods to assess thyroid hormones, as detailed in Table 1, which included structural evaluation via ultrasonographic examination [15]. Five studies analyzed TSH levels, with only one [23] examining PTH levels. Baum, Börner [21] conducted multiple tests, including immunological markers such as thyroglobulin and microsomal thyroid antibody. Table 1 presents all the significant information.

**Table 1:** Data extraction from the elected studies.

Author (year)	Study Type	Participants				Exposure to F (drinking water)	Diagnostic Method		Results
		Source	Sample size (n)		Age		Fluoride levels	Thyroid function	
**Leone et al. (1964)**	Cross-sectional	New York, EUA; Crisfield, Maryland, EUA	Low F levels: 109 High F levels: 106		18–60	Low F levels: 0.09 mg/L High F levels: 3.48mg/L	Source: Water supply Method: Not informed	Sample: Serum Method: Protein Bound Iodine (PBI) test	No significant changes were found in PBI between both groups.
**Baum et al. (1981)**	Cross-sectional	Nuremberg and Wendelstein, Germany	Low F levels: 17 High F levels: 26		Not informed	Low F levels: 0.1 mg/L– 0.2 mg/L High F levels: ~ 3 mg/L	Source: Water supply Method: Not informed	Sample: Not informed Methods: T_3_ Uptake, RIA-T_3_, rT_3_, hTg, TSH, T_4_-Index, Thyroglobulin and Microsomal Thyroid Antibody.	No significant changes were found between both groups.
**Wang et al (2001)**	Cross-sectional	Qingyun County, China	Low F levels: 31–33 High F levels: 27–29	8–12		Low F levels: 0.5 mg/L High F levels: 2.97 mg/L	Source: Water supply Method: Fluorine ion selective electrode	Sample: Serum Method: Thyroid iodine-131 uptake, T3 and T4 (RIA), TSH (IRMA).	Thyroid iodine-131 uptake was lower and TSH levels were higher in participants exposed to high F levels
**Kutlucan et al. (2013)**	Cross-sectional	Isparta, Turkey	Low F levels: 298 High F levels: 261		10–15	Low F levels: 0.19 mg/L High F levels: 2.8 mg/L– 4.6 mg/L	Source: Water supply Method: Not informed	Sample: Urine; Organ volume. Method: Iodine elimination by urine spot test; ultrasonographic evaluation (thyroid gland)	The higher levels of fluoride were related to a higher echo body index of the thyroid gland.
**Khandare et al (2018)**	Cross-sectional	Telangana, India	Low F levels: 645 High F levels: 962		8–14	Low F levels: 0.87 mg/L; High F levels: 2.53 mg/L –3.77 mg/L	Samples: Water supply; Method: Fluoride-specific electrode (Orion 9609).	Sample: Serum Method: T3, T4 and TSH (RIA); PTH (IRMA).	Individuals from higher F level areas had lower T3 levels, and higher T4, TSH and PTH.
**Zulfiqar et al. (2019)**	Cross-sectional	Pakistan	Low F levels: 60 High F levels: 74		8–15	Low F levels: 0.54 mg/L; High F levels: 4.66 mg/L	Sample: Water supply; Method: Fluoride ion selective electrode	Sample: Serum Method: FT4, T3, TSH by (IRMA and RIA)	TSH levels were significantly higher in individuals from the area with high F levels.
**Zulfiqar et al. (2020)**	Cross-sectional	Talab Saraiand and Ottawa, Pakistan	Low F levels: 60 High F levels: 130		7–18	Low F levels: 0.54 mg/L High F levels: 6.23 mg/L	Samples: Water supply; Method: Fluoride-selective electrode	Samples: Serum FT4 and FT3 (RIA); TSH (IRMA)	TSH and FT3 showed difference in the comparison between groups; No correlation was observed in FT4 and FT3, with serum and water F levels; The was a strong correlation between TSH and water F levels.

### Risk of bias

Two studies were considered high risk in the bias due to confounding domain and due to some concerns regarding potential risk of bias arising from measurement of the outcome [20, 21]. Four included studies were considered low risk [15, 22–24]. However, one study was considered some concerns in the bias due to potential risk of bias from measurement of the exposure [25]. (Table 2).

**Table 2:** Risk of Bias among included studies according to ROBINS-E.

Studies	Risk of bias due to confounding	Risk of bias arising from measurement of the exposure	Risk of bias in selecting participants in the study	Risk of bias due to post-exposure interventions	Risk of bias due to missing data	Risk of bias arising from measurement of the outcome	Risk of bias in selection of the reported result	Study-level RoB Judgment
**Leone et al. 1964**	High	Low	Low	Low	Low	Some concerns	Low	High
**Baum et al. 1981**	High	Low	Low	Low	Low	Some concerns	Low	High
**Wang et al. 2001**	Low	Low	Low	Low	Low	Low	Low	Low
**Kutuclan 2013**	Low	Low	Low	Low	Low	Low	Low	Low
**Khandare et al. 2018**	Low	Low	Low	Low	Low	Low	Low	Low
** Zulfiqar et al. 2019**	Low	Low	Low	Low	Low	Low	Low	Low
** Zulfiqar et al. 2020**	Low	Some concerns	Low	Low	Low	Low	Low	Some concerns

### Level of evidence

Given the variety of age groups and the diverse methods employed to assess thyroid function, four parameters were evaluated: alterations in T3, T4, TSH, and PTH levels in response to both low and high F exposure (Table 3). Furthermore, a significant risk was identified owing to a high bias risk in one of the incorporated studies [20]. The overall confidence in the evidence was deemed low for all outcomes (Tables 3).

**Table 3:** The certainty of evidence.

High fluoride levels compared to Low fluoride levels for thyroid gland in adults						
**Patient or population:** thyroid gland in humans **Setting:** **Intervention:** High fluoride levels **Comparison:** Low fluoride levels						
**Outcome** **№ of participants** **(studies)**	**Relative effect (95% CI)**	**Anticipated absolute effects (95% CI)**			**Certainty**	**What happens**
				**Difference**		
Changes in the Triiodothyronine (T3) hormone № of participants: 2036 (5 non-randomised studies)	not estimable	0.0%	**0.0%** (0 to 0)	**0.0% fewer** (0 fewer to 0 fewer)	⨁⨁◯◯ Low^a^	Individuals from higher fluoride level areas had lower T3 levels
Changes in the Thyroxine (T4) hormone № of participants: 2036 (5 non-randomised studies)	not estimable	0.0%	**0.0%** (0 to 0)	**0.0% fewer** (0 fewer to 0 fewer)	⨁⨁◯◯ Low^b^	High fluoride levels have shown changes in the T4 levels of adults
Changes in the Thyroid-stimulating hormone (TSH) № of participants: 2036 (5 non-randomised studies)	not estimable	0.0%	**0.0%** (0 to 0)	**0.0% fewer** (0 fewer to 0 fewer)	⨁⨁◯◯ Low^a^	The TSH levels in children were higher in participants exposed to high F levels
Serum levels of iodine-bound protein (PBI) № of participants: 215 (1 non-randomised study)	not estimable	0.0%	**0.0%** (0 to 0)	**0.0% fewer** (0 fewer to 0 fewer)	⨁◯◯◯ Very low^b^	The fluoride was not able to modulate protein-bound iodine levels

*The risk in the intervention group (and its 95% confidence interval) is based on the assumed risk in the comparison group and the **relative effect** of the intervention (and its 95% CI).**CI:** confidence interval

**GRADE Working Group grades of evidenceHigh certainty:** we are very confident that the true effect lies close to that of the estimate of the effect.**Moderate certainty:** we are moderately confident in the effect estimate: the true effect is likely to be close to the estimate of the effect, but there is a possibility that it is substantially different.**Low certainty:** our confidence in the effect estimate is limited: the true effect may be substantially different from the estimate of the effect.**Very low certainty:** we have very little confidence in the effect estimate: the true effect is likely to be substantially different from the estimate of effect.

## Discussion

This systematic review collates findings from primary studies that utilized WHO guidelines to determine low and high F levels, with the aim of assessing the association between F exposure and thyroid dysfunction. Out of the seven cross-sectional studies examined, five identified an association between F exposure and certain impairments of the thyroid gland. However, owing to significant biases in sample selection and analytical methodologies, a definitive conclusion regarding this association cannot be confidently drawn.

Numerous *in vivo* and observational studies have explored the impact of F on the thyroid, considering variables such as species, age, sex, F dosage, and exposure duration [26–29]. Research suggests that F can influence thyroid function by inhibiting the sodium/iodide symporter, a crucial component for iodine uptake from the bloodstream into thyrocytes and its subsequent incorporation into thyroglobulin [30]. Given this information, it is crucial to note that the thyroid gland is particularly susceptible to interactions with F. While F is primarily deposited in mineralized tissues, studies indicate that the concentration of F in thyroid tissue often exceeds levels found in other soft tissues, excluding the kidney [31]. Furthermore, the presence of F in thyroid tissues disrupts the normal operation of thyroid hormones and induces morphofunctional changes [32].

Hormonal levels are regulated through feedback mechanisms, which maintain hormonal balance [33]. Disturbances in hormone concentrations necessitate laboratory tests for monitoring and diagnosing changes in TSH, T3, and T4 levels (both total and free). Immunoassays from various generations, such as radio-immunoassays (RIA), immunoradiometric assay (IRMA), Enzyme Linked Immuno Sorbent Assay (ELISA) and Immunochemiluminometric assays (ICMA) are the most frequently used and validated methods in the literature for sensitive measurement of these hormonal levels [34, 35]. However, the sensitivity and specificity of different methods vary, complicating the comparison of quantitative results across studies. In addition to the work of [20–22] have utilized other valuable markers. Baum, Börner [21] evaluated thyroglobulin and microsomal thyroid antibody. The former is a marker for the precursor of thyroid hormones and is commonly assessed in cases of neoplasms, Grave’s disease, and iodine deficiency. The latter is primarily investigated in autoimmune conditions [36, 37]. Wang, Wang [22], on the other hand, assessed thyroid iodine-131 uptake. This test is performed following the administration of a radiotracer (iodine-131), enabling an estimation of the amount of radiotracer absorbed by the thyroid.

This systematic review included two additional hormones, PTH and TSH, which, despite not originating from the thyroid glands, play a significant role in body homeostasis and thyroid function. PTH is produced in the parathyroid gland and primarily regulates mineral metabolism. Conversely, TSH is produced by the anterior pituitary gland and facilitates the synthesis and release of thyroid hormones. Clinically, elevated TSH levels often correlate with reduced T3 and T4 production, indicating a response to hormone deficiency and a need for increased production and release. This pattern was observed in studies by Khandare, Validandi [23] and [24, 25], although [22] reported no such correlation. Moreover, while an increase in PTH is relevant, it does not directly influence thyroid functions.

F has been found to modify the production of thyroid hormones at varying exposure levels, thereby acting as an endocrine disruptor and impairing thyroid function [9]. Several authors have proposed that a high concentration of F in drinking water could predict the prevalence of hypothyroidism [38, 39]. However, most studies have not provided a clear diagnosis, merely reporting observed differences between groups. It is important to note that the majority of the included studies focused on the effects of F on children and young people, who exhibited no significant changes in the thyroid gland. This observation could be linked to the duration of exposure, as the effects of F are known to be time-dependent [40, 41].

The participant characteristics mentioned above warrant emphasis owing to the clinical differences observed across age and sex. For instance, Leone, Leatherwood [20] included individuals aged 18 to 60 years but did not distinguish between male and female participants. Furthermore, the remaining studies that incorporated children and adolescents in their cohorts failed to stratify participants by age groups, which would allow for evaluation of results against appropriate reference values. The only exception was Kutlucan, Koroglu [15], that assessed morphological parameters using ultrasonographic examination. Given the time period in which most of these articles were conducted, it is plausible that different reference ranges were used for each hormone rate. This methodological issue undermines the clinical interpretation of the results. Currently, reference values can be readily accessed through the reports of the American Association of Clinical Endocrinology and the European Society of Endocrinology, both of which offer updated guidelines on diagnostic methods and criteria.

To evaluate endogenous hormone production, numerous laboratory tests have been developed over the years. The oldest article included in this systematic review illustrates changes in the methods employed to determine thyroid function over time, when compared to the other articles in our review. Leone, Leatherwood [20] utilized the protein bound iodine test, which is based on the proportionality between the amount of iodine precipitated from plasma proteins and thyroxine levels [42]. Conversely, the development and clinical application of immunoassays began after the 1970s. These tests incorporate the use of radioisotopes and antibodies to determine hormone concentrations [43]. From this period onwards, none of the subsequent articles employed older methodologies to assess outcomes. For instance, Baum, Börner [21] utilized various immunoassays, a trend that continued in later studies.

One of the studies included in this review Kutlucan, Koroglu [15] evaluated the impact of F on thyroid tissue volume, concluding that F can independently increase thyroid size, irrespective of iodine. Previous literature has reported morphological changes such as mild atrophy of the follicular epithelium [44], distended endoplasmic reticulum in follicular cells [45], and alterations indicative of hormonal hypofunction [31, 46]. However, the methods used to diagnose thyroid function and the age groups of the populations studied varied across these studies. Consequently, the evidence level was classified as low, as per the GRADE tool. The GRADE assessment classifies the level of certainty into four categories: very low, low, moderate, and high [47]. The low classification assigned in this study is attributed to the clinical heterogeneity among the studies and the nature of the included studies, all of which are observational. This methodological variability precluded the possibility of conducting a meta-analysis.

The limitations of this systematic review stem from methodological issues observed in the studies, particularly those pertaining to the sampling process, including the lack of control of potential confounding factors, sample calculation or randomization. Also, lack of clarity on how to the Fluoride levels as well as how the thyroid dysfunction was assessed on some studies was observed. These limitations heighten the risk of bias in the studies and diminish the inferential power of the findings. Consequently, we advocate for investigations employing robust methodology to evaluate the association between F intake and thyroid dysfunction. It is crucial to recommend new studies with more robust methodologies concerning participant grouping, laboratory methods for assessing thyroid hormones, and determining F levels in drinking water. Furthermore, future studies should establish inclusion criteria that highlight the duration of previous F exposure, the varying ages of children, adolescents, and adults, as well as the presence of hormonal factors in adult women, such as menopause and pregnancy.

## Conclusion

The primary articles’ evidence summary suggests an association between high F level exposure and thyroid function modulation. However, the quality and certainty of this evidence is not of sufficient quality to draw such strong conclusion, particularly when the methodological structure of the articles is thoroughly evaluated. Furthermore, highlight the importance of more human studies being carried out to better elucidate the understanding of fluoride as an environmental pollutant and its repercussions on human health in regions with high fluoride levels.

## Supporting information

S1 Table PRISMA 2020 checklist. (DOCX)

S2 Table List of MeSH and entry terms which composed the search strategy following the PECO acrostic. (DOCX)
