# Supplementary material for: Is there any association between fluoride exposure and thyroid function modulation? A systematic review
Source: PLoS One. 2024 Apr 9;19(4):e0301911. doi: 10.1371/journal.pone.0301911 (PMC11003687; doi:10.1371/journal.pone.0301911)
Supplement: S2 Table — (DOCX) [file pone.0301911.s002.docx]

**Supplementary Table S2.** List of MeSH and Entry Terms which composed the search strategy following the PECO acrostic.

| **P** | **Humans** | Homo sapiens |
| --- | --- | --- |
|  |  | Man (Taxonomy) |
|  |  | Man, Modern |
|  |  | Modern Man |
|  |  | Human |
|  |  |  |
|  | **Adult** | Adults |
|  |  |  |
|  | **Adolescent** | Adolescents |
|  |  | Adolescence |
|  |  | Teens |
|  |  | Teen |
|  |  | Teenagers |
|  |  | Teenager |
|  |  | Youth |
|  |  | Youths |
|  |  | Adolescents, Female |
|  |  | Adolescent, Female |
|  |  | Female Adolescent |
|  |  | Female Adolescents |
|  |  | Adolescents, Male |
|  |  | Adolescent, Male |
|  |  | Male Adolescent |
|  |  | Male Adolescents |
|  |  |  |
|  | **Child** | Children |
|  |  |  |
|  | **Infant** | Infants |
|  |  |  |
|  | **Aged** | Elderly |
|  |  |  |
|  | **Men** | Boys |
|  |  |  |
|  |  |  |
|  | **Women** | Girl |
|  |  | Woman |
|  |  | Women's Groups |
|  |  | Women Groups |
|  |  | Women's Group |
| **E** | **Fluorides** | Fluoride |
|  |  |  |
|  | **Fluorine** | Fluorine-19 |
|  |  | Fluorine 19 |
|  |  |  |
|  | **Fluoridation** | Milk Fluoridation |
|  |  | Fluoridation, Milk |
|  |  | Water Fluoridation |
|  |  | Fluoridation, Water |
|  |  | Salt Fluoridation |
|  |  | Fluoridation, Salt |
|  |  |  |
|  | **Fluoride Poisoning** | Poisoning, Fluoride |
|  |  | Fluoride Poisonings |
|  |  | Poisonings, Fluoride |
|  |  |  |
|  | **Fluorine Compounds** | Compounds, Fluorine |
| **O** | **Thyroid Gland** | Gland, Thyroid |
|  |  | Glands, Thyroid |
|  |  | Thyroid Glands |
|  |  | Thyroid |
|  |  | Thyroids |
|  |  |  |
|  | **Thyroid Diseases** | Disease, Thyroid |
|  |  | Diseases, Thyroid |
|  |  | Thyroid Disease |
|  |  |  |
|  | **Hyperthyroidism** | Hyperthyroid |
|  |  | Hyperthyroids |
|  |  | Primary Hyperthyroidism |
|  |  | Hyperthyroidism, Primary |
|  |  |  |
|  | **Hypothyroidism** | Hypothyroidisms |
|  |  | Primary Hypothyroidism |
|  |  | Hypothyroidism, Primary |
|  |  | Primary Hypothyroidisms |
|  |  | Thyroid-Stimulating Hormone Deficiency |
|  |  | Deficiency, Thyroid-Stimulating Hormone |
|  |  | Hormone Deficiency, Thyroid-Stimulating |
|  |  | Thyroid Stimulating Hormone Deficiency |
|  |  | Thyroid-Stimulating Hormone Deficiencies |
|  |  | TSH Deficiency |
|  |  | Deficiency, TSH |
|  |  | TSH Deficiencies |
|  |  | Secondary Hypothyroidism |
|  |  | Hypothyroidism, Secondary |
|  |  | Secondary Hypothyroidisms |
|  |  | Central Hypothyroidism |
|  |  | Central Hypothyroidisms |
|  |  | Hypothyroidism, Central |
|  | **Thyroid Hormones** | Hormones, Thyroid |
|  |  | Thyroid Hormone |
|  |  | Hormone, Thyroid |
|  | **Calcitonin** | Calcitrin |
|  |  | Thyrocalcitonin |
|  |  | Calcitonin(1-32) |
|  |  | Ciba 47175-BA |
|  |  | Ciba 47175 BA |
|  |  | Ciba 47175BA |
|  |  | Eel Calcitonin |
|  |  | Calcitonin, Eel |
|  | **Dextrothyroxine** | D-T4 Thyroid Hormone |
|  |  | D-Thyroxine |
|  |  | D Thyroxine |
|  |  | Choloxin |
|  |  | Dextrothyroxine Sodium |
|  |  | Sodium Dextrothyroxine |
|  | **Diiodotyrosine** | Iodogorgoic Acid |
|  |  | Acid, Iodogorgoic |
|  | **Monoiodotyrosine** | Iodotyrosine |
|  | **Thyronines** | Thyronine |
|  | **Diiodothyronines** | |
|  | **Triiodothyronine** | T3 Thyroid Hormone |
|  |  | Thyroid Hormone, T3 |
|  |  | Liothyronine |
|  |  | 3,3',5-Triiodothyronine |
|  |  | Liothyronine Sodium |
|  |  | Cytomel |
|  | **Triiodothyronine, Reverse** | Reverse Triiodothyronine |
|  |  | 3,3,5-Triiodothyronine |
|  |  | Reverse T3 Thyroid hormone |
|  |  | 3,3',5'-Triiodothyronine |
|  |  | 3,3,5 Triiodothyronine |
|  | **Thyroxine** | O-(4-Hydroxy-3,5-diiodophenyl)-3,5-diiodotyrosine |
|  |  | Thyroxin |
|  |  | 3,5,3',5'-Tetraiodothyronine |
|  |  | T4 Thyroid Hormone |
|  |  | Thyroid Hormone, T4 |
|  |  | Synthrox |
|  |  | Levothyroxine Sodium |
|  |  | Sodium Levothyroxine |
|  |  | Thyrax |
|  |  | Tiroidine |
|  |  | Tiroxina Leo |
|  |  | Unithroid |
|  |  | Eferox |
|  |  | Eltroxin |
|  |  | Thevier |
|  |  | Eltroxine |
|  |  | Euthyrox |
|  |  | Eutirox |
|  |  | L-Thyrox |
|  |  | L Thyrox |
|  |  | L-Thyroxin beta |
|  |  | L Thyroxin beta |
|  |  | L-Thyroxin Henning |
|  |  | L Thyroxin Henning |
|  |  | Levothyroxine |
|  |  | O-(4-Hydroxy-3,5-diiodophenyl) 3,5-diiodo-L-tyrosine |
|  |  | L-Thyroxine |
|  |  | L Thyroxine |
|  |  | L-3,5,3',5'-Tetraiodothyronine |
|  |  | Levoxine |
|  |  | Levoxyl |
|  |  | Lévothyrox |
|  |  | L-Thyroxine Roche |
|  |  | L Thyroxine Roche |
|  |  | Levo-T |
|  |  | Levo T |
|  |  | Levothroid |
|  |  | Novothyral |
|  |  | Berlthyrox |
|  |  | Dexnon |
|  |  | Novothyrox |
|  |  | Oroxine |
|  |  | Synthroid |
|  |  | Levothyroxin Deladande |
|  |  | Levothyroxin Delalande |
|  |  | Levothyroid |
|  | **Thyrotropin** | TSH (Thyroid Stimulating Hormone) |
|  |  | Thyrotrophin |
|  |  | Thyroid-Stimulating Hormone |
|  |  | Hormone, Thyroid-Stimulating |
|  |  | Thyroid Stimulating Hormone |
|  |  | Thyreotropin |
